# A Rare Presentation of Pulmonary Transthyretin Amyloidosis With Persistent Ground-Glass Opacity Diagnosed by Transbronchial Lung Biopsy

**DOI:** 10.7759/cureus.104269

**Published:** 2026-02-25

**Authors:** Kazuki Uchida, Kazunori Tobino

**Affiliations:** 1 Respiratory Medicine, Iizuka Hospital, Iizuka, JPN

**Keywords:** ground-glass opacity, interstitial lung disease, pulmonary amyloidosis, transbronchial lung biopsy, transthyretin amyloidosis

## Abstract

Pulmonary-dominant transthyretin (TTR) amyloidosis is an uncommon condition. Its presentation with persistent ground-glass opacity (GGO) and infiltrates mimics common respiratory diseases, causing diagnostic delays. Here, we report a case of an 80-year-old male patient with a five-year history of GGO refractory to antibiotics and steroids. Endobronchial ultrasound-guided transbronchial lung biopsy confirmed TTR amyloidosis. Investigations revealed subclinical cardiac involvement, supporting systemic amyloid TTR (ATTR) with a pulmonary-dominant phenotype. Treatment with tafamidis was initiated; however, pulmonary lesions remained radiologically stable at two months and at one year of follow-up. Given that tafamidis is expected to stabilize TTR and slow disease progression rather than rapidly regress established deposits, short-term radiologic stability should be interpreted cautiously. This case demonstrates the diagnostic utility of minimally invasive bronchoscopic techniques for unexplained lung opacities and highlights the need for further evidence regarding the clinical course and treatment response of pulmonary manifestations of ATTR.

## Introduction

Transthyretin (TTR) amyloidosis is a systemic disorder caused by the deposition of insoluble TTR fibrils within tissues, manifesting as restrictive cardiomyopathy or progressive polyneuropathy. Historically, significant pulmonary involvement has been regarded as rare [1]. However, emerging evidence suggests that pulmonary TTR deposition is under-recognized [2,3]. The pulmonary spectrum is broad, ranging from incidentally detected nodules to diffuse alveolar-septal amyloidosis [3-6]. Because radiologic appearances are often non-specific, mimicry with infection, organizing pneumonia, or malignancy can delay diagnosis and create pitfalls. Definitive confirmation relies on Congo red staining-showing apple-green birefringence under polarized light-and amyloid typing, commonly by immunohistochemistry (IHC), to differentiate TTR from other precursors such as immunoglobulin light chains (AL) [5,6].

Importantly, TTR amyloidosis comprises wild-type amyloid TTR (ATTR) (ATTRwt) and hereditary ATTR (hATTR). Definitive distinction requires TTR gene sequencing; when genetic testing is unavailable, the genetic subtype should be described as indeterminate and interpreted with appropriate caution. Therapeutic advances, including TTR stabilizers and gene-silencing agents, have improved outcomes in systemic disease, but their effects on established pulmonary deposits remain uncertain [3,5,7]. Here, we report a case in which endobronchial ultrasound-guided transbronchial lung biopsy using a guide sheath (EBUS-GS-TBLB) provided a definitive diagnosis, illustrating a safe alternative to surgical biopsy [5], and we document longitudinal radiologic stability of pulmonary findings under tafamidis therapy.

## Case presentation

**Figure 1:**
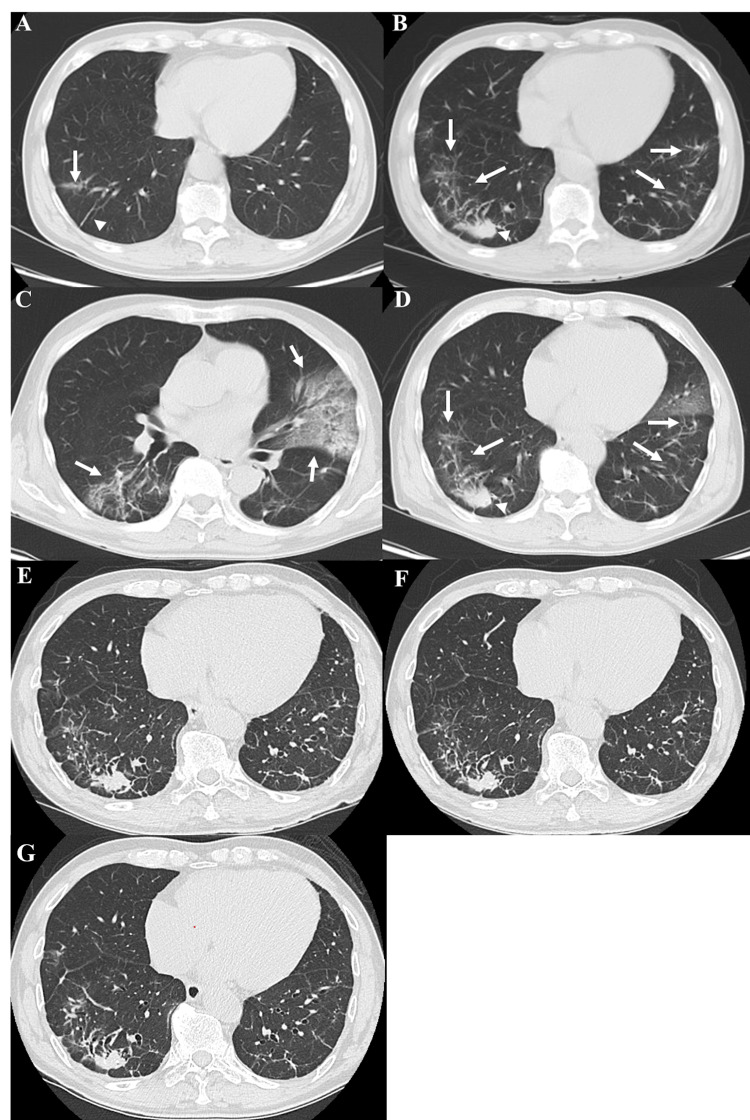
Chest CT images. (A) A CT scan obtained five years ago shows a slight GGO (arrow) and a linear shadow (arrowhead) in the right lower lobe. (B) CT scan performed one month before presentation shows GGOs in both lower lobes (arrows) of the lungs and a nodular shadow in the right lower lobe (arrowhead). (C, D) CT scan on admission showed a new GGO in the left upper lobe (C, arrow), while the previously existing GGOs and nodules in the lower lobes remained largely unchanged (D, arrows and arrowhead, respectively). (E) CT scan performed at the initiation of tafamidis meglumine therapy. (F) Follow-up CT scan after two months of treatment showing stability of the opacities in the right lower lobe. (G) Follow-up CT scan after one year of tafamidis therapy showing continued radiologic stability of the pulmonary opacities. GGO: ground-glass opacity

An 80-year-old man was referred for evaluation of a year-long, gradually worsening cough with fever despite multiple empiric treatments at a local clinic. Five years earlier, routine screening CT had revealed ground-glass opacity (GGO) and a linear shadow in the right lower lobe (Figure 1A), unchanged on follow-up six months later. He was a never-smoker, denied dust exposure, and had no family history of amyloidosis. Six months before admission, he was diagnosed with COVID-19 and received antibiotics and steroids; his cough persisted. One month before admission, CT showed new GGOs in both lower lobes and a nodular opacity in the right lower lobe (Figure 1B).

On admission, vital signs were stable, and oxygen saturation was 97% on room air. Coarse crackles were audible over the left lung, and C-reactive protein was markedly elevated at 12.77 mg/dL. Chest CT demonstrated new GGOs in the left upper and right lower lobes (S6) (Figure 1C), whereas the previously identified GGOs and nodular shadows in both lower lobes were unchanged (Figure 1D). Intravenous ceftriaxone resulted in the resolution of the left-sided opacity, but the right-sided lesion persisted.

**Figure 2:**
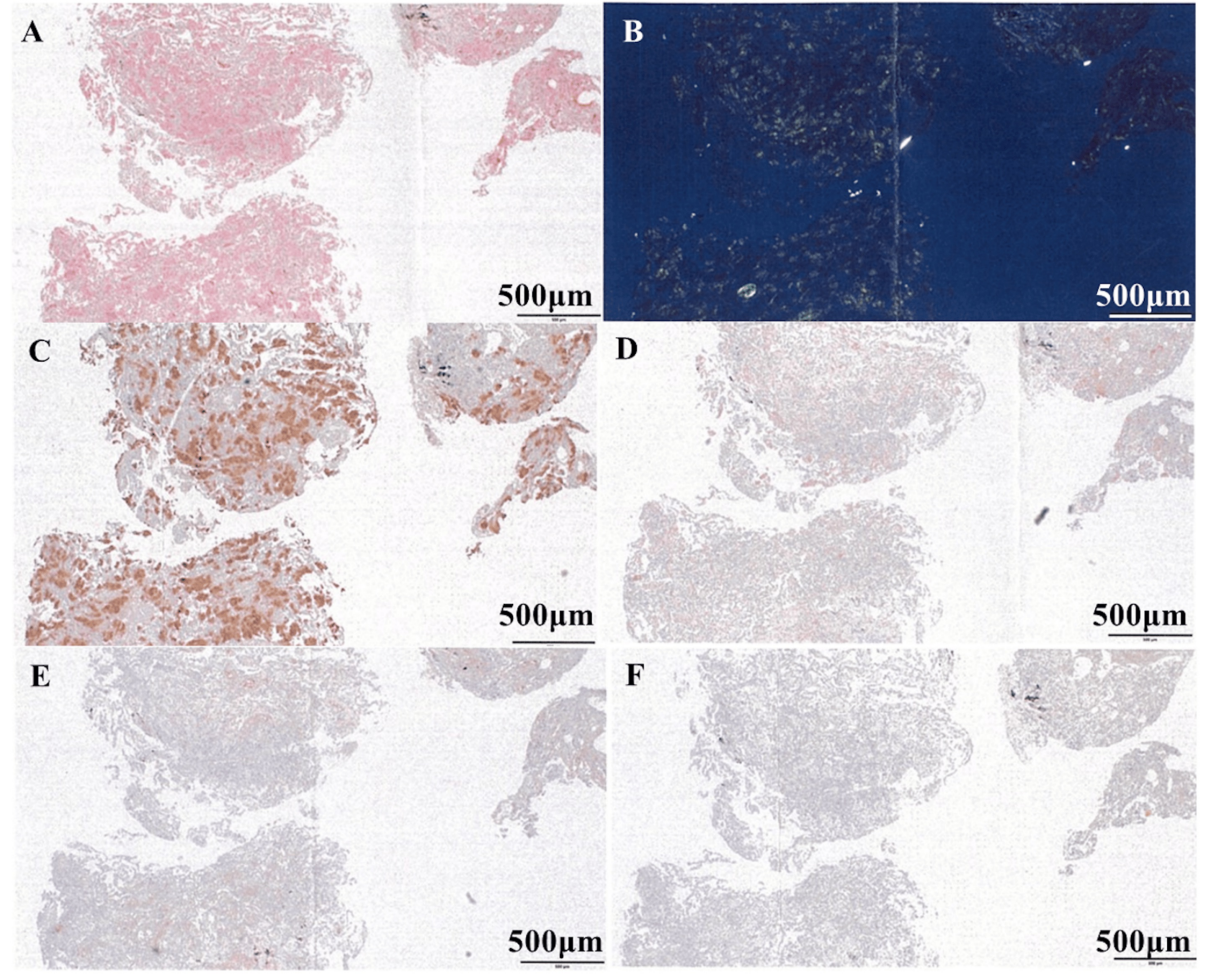
Histopathological findings of transbronchial lung biopsy. (A) Congo red staining reveals pink, amorphous amyloid deposits within the alveolar septa. (B) Under polarized light, the same deposits show pathognomonic apple-green birefringence. (C) Immunohistochemistry demonstrates strong positivity for transthyretin (TTR). (D-F) Stains for AL-kappa, AL-lambda, and serum amyloid A (AA) are negative, confirming the TTR subtype (scale bar = 500 µm).

To investigate the non-resolving opacity, EBUS-GS-TBLB targeted to the right lower lobe was performed. Histology revealed amorphous deposits expanding alveolar septa that stained with Congo red (Figure 2A) and produced apple-green birefringence on polarized light (Figure 2B), confirming amyloid. IHC was strongly positive for TTR (Figure 2C) and negative for AL-κ, AL-λ, and AA (Figures 2D-2F), supporting ATTR. Mass spectrometry-based amyloid typing was not available at our institution.

Laboratory evaluation for monoclonal gammopathy/plasma cell dyscrasia showed hemoglobin 14.4 g/dL, creatinine 1.03 mg/dL (estimated glomerular filtration rate (eGFR) 53.40 mL/min/1.73 m²), and calcium 9.3 mg/dL. Urinalysis showed no hematuria and negative dipstick protein; quantitative urine protein was <4.3 mg/dL with a urine protein/creatinine ratio < 0.07. Serum protein electrophoresis (performed at the time of pathological typing) showed no M-spike and the following fractions: albumin 3.80 g/dL (59.3%), α1-globulin 0.29 g/dL (4.6%), α2-globulin 0.65 g/dL (10.2%), β1-globulin 0.35 g/dL (5.5%), β2-globulin 0.28 g/dL (4.4%), and γ-globulin 1.02 g/dL (16.0%); β2-microglobulin was 2.0 mg/L. Serum immunofixation electrophoresis was negative. Serum free light chain assay showed κ 27.9 and λ 23.0 with a κ/λ ratio of 1.21. Urine Bence Jones protein was negative. The patient reported no bone pain; however, imaging evaluation for bone lesions was not performed.

**Figure 3:**
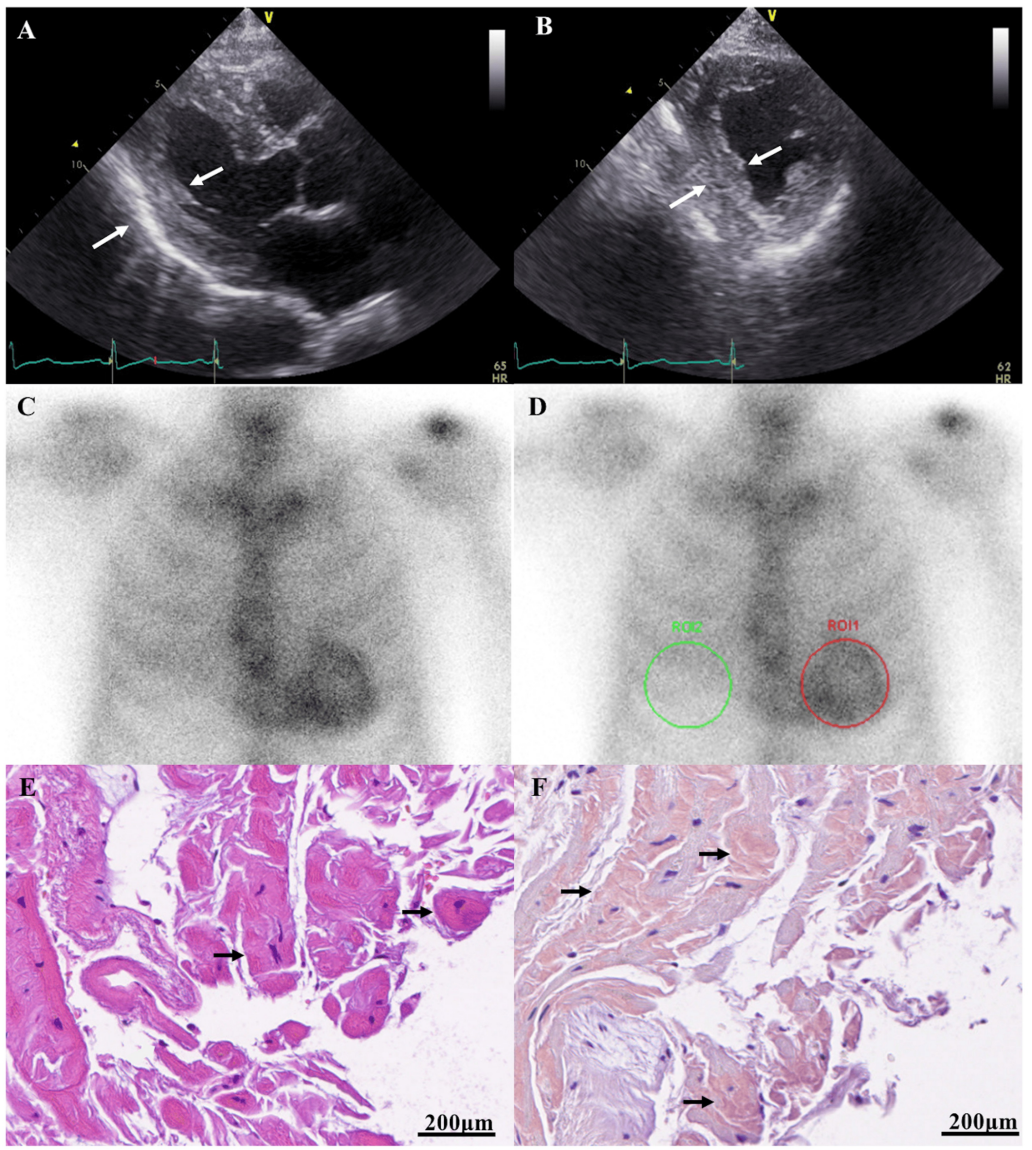
Multimodality evaluation of cardiac amyloidosis. (A, B) Transthoracic echocardiography (parasternal long-axis and short-axis views) demonstrates marked symmetrical thickening of the left ventricular walls (arrows) and a granular sparkling myocardial texture. (C, D) Technetium-99m pyrophosphate scintigraphy shows intense myocardial tracer uptake (C), corresponding to a Perugini grade of 3. For quantitative analysis, regions of interest (ROIs) are placed over the heart (D, red circle) and contralateral lung (D, green circle) to yield a heart-to-contralateral lung (H/CL) ratio of 1.935. (E, F) Histopathological findings of the endomyocardial biopsy. (E) Hematoxylin and eosin staining showed interstitial deposition of an amorphous eosinophilic material (arrows). (F) Congo red staining confirms that these interstitial deposits are amyloid (arrows) (scale bar = 200 µm).

Systemic evaluation for cardiac involvement identified symmetric thickening of the interventricular septum (IVS) and left-ventricular posterior wall (LVPW) with a granular sparkling myocardial appearance on transthoracic echocardiography (Figures 3A, 3B). Baseline B-type natriuretic peptide (BNP) was 126.3 pg/mL. Baseline echocardiography showed IVS 13 mm, LVPW 12 mm, and left-ventricular ejection fraction (LVEF) 45%. Technetium-99m pyrophosphate scintigraphy demonstrated intense cardiac uptake (visual score grade 3) with a heart-to-contralateral ratio of 1.935 (Figures 3C, 3D). Endomyocardial biopsy revealed cardiomyocyte hypertrophy and atrophy with extensive interstitial deposition of amorphous Congo-red-positive material, confirming cardiac amyloidosis (Figures 3E, 3F).

Based on these findings, a final diagnosis of systemic ATTR with a pulmonary-dominant presentation and subclinical cardiac involvement was made. TTR gene sequencing was not performed because it was not available at our institution; thus, the genetic subtype (ATTRwt vs. hATTR) remained indeterminate, although the late-onset phenotype was clinically suggestive of ATTRwt. Despite the cardiac findings, his New York Heart Association class was I. Tafamidis meglumine was initiated. A baseline CT at therapy start is shown in Figure 1E; at two months, symptoms were stable and pulmonary opacities were unchanged on repeat CT (Figure 1F). A further follow-up CT at one year also showed no substantial change, demonstrating continued radiologic stability (Figure 1G).

To facilitate longitudinal comparison, we performed a semi-quantitative visual assessment of GGO extent at three standardized axial levels (upper: aortic arch; mid: carina; lower: above the diaphragm). The estimated percentage of lung area involved by GGO at each level across time points is summarized in Table 1. This assessment was performed once by a single evaluator. Cardiology follow-up was conducted at another institution; a referral letter stated that cardiac findings were unchanged, but quantitative follow-up parameters (e.g., serial BNP/global longitudinal strain (GLS) or detailed echocardiographic measurements) were not available in our records.

**Table 1:** Semi-quantitative longitudinal assessment of ground-glass opacity (GGO) extent on chest CT. Upper slice: aortic arch level; mid slice: carina level; lower slice: 1-2 cm above the right hemidiaphragm. Values indicate the visually estimated percentage of lung parenchyma involved by GGO, assessed in 5% increments by a single evaluator in a single pass using consistent lung window settings (e.g., window length (WL) −600 and window width (WW) 1,500 when available). This descriptive approach is not a validated quantitative CT method; minor differences across time points should be interpreted in the context of interscan and visual-estimation variability.

Time point	Upper slice (%)	Mid slice (%)	Lower slice (%)
Five years before presentation	0	0	5
Prior clinic CT	5	15	15
At first visit (admission CT)	0	15	20
Immediately before tafamidis	5	10	10
2 months after tafamidis	0	10	15
1 year after tafamidis	5	15	10

## Discussion

This case demonstrates a rare systemic disease presenting with a common radiologic pattern and underscores the utility of a minimally invasive diagnostic strategy. Because GGOs and small nodules are non-specific, pulmonary amyloidosis is often not considered initially and may be discovered incidentally, including in the background lung from cancer resections [3]. Reported pulmonary phenotypes include nodules that mimic malignancy [4] and diffuse GGO resembling organizing pneumonia, as suspected initially here and described elsewhere [5]. Accordingly, in older adults with persistent, unexplained infiltrates-especially when lesions wax and wane against a background of indolent abnormalities-ATTR should be included in the differential diagnosis.

With respect to tissue diagnosis, while surgical lung biopsy has been a reference standard for diffuse parenchymal lung disease, risk-benefit considerations in elderly patients favor bronchoscopic strategies when feasible. Evidence supports transbronchial biopsy for pulmonary amyloidosis [8]. The present case shows that EBUS-GS-TBLB can yield sufficient tissue for Congo red confirmation and subtype-specific IHC, allowing a conclusive diagnosis of ATTR without surgical morbidity, in line with prior reports [5]. Management must also account for systemic involvement. Detection of subclinical cardiomyopathy alongside pulmonary-dominant symptoms highlights the need for comprehensive multiorgan assessment at baseline.

Tafamidis is a TTR tetramer stabilizer expected to slow progression by preventing further deposition rather than inducing rapid regression of established deposits. Therefore, radiologic stability of pulmonary lesions at two months-and continued stability at one year-should not be interpreted as treatment failure. Instead, it highlights the limited published evidence specifically addressing pulmonary involvement in ATTR and the need for longer longitudinal observation to clarify pulmonary trajectories under therapy.

Limitations

This report describes a single case and has limited longitudinal cardiopulmonary biomarker data beyond baseline assessment (including BNP 126.3 pg/mL). Definitive genetic subtype classification (ATTRwt vs. hATTR) was not possible because TTR gene sequencing was unavailable at our institution. Mass spectrometry-based proteomic amyloid typing (e.g., laser microdissection with liquid chromatography-tandem mass spectrometry (LC-MS/MS)) was also not available, and amyloid subtype determination relied on IHC in a transbronchial specimen. IHC-based typing may be limited by antibody specificity, background staining, and potential misclassification; therefore, results should be interpreted in conjunction with clinical and systemic evaluation. AL amyloidosis was systematically screened and was not supported by negative serum immunofixation, a non-abnormal serum free light chain κ/λ ratio, and negative urine Bence Jones protein, together with no M-spike on serum protein electrophoresis. Imaging evaluation for bone lesions was not performed. Serial cardiac biomarkers and echocardiographic parameters at follow-up were not available in our records because follow-up was conducted at another institution; only a referral letter stated that findings were unchanged. Finally, the semi-quantitative longitudinal GGO assessment (Table 1) was a single-rater visual estimate performed once without formal validation; thus, small fluctuations across time points may reflect interscan and visual-estimation variability rather than true interval change.

## Conclusions

Pulmonary ATTR may present insidiously with persistent GGOs and nodules in older adults and should be considered in unresolved lung disease. EBUS-GS-TBLB enables definitive, minimally invasive diagnosis through Congo red staining and IHC typing and should be considered early when appropriate. In this single case with constrained subtype classification and limited longitudinal objective data, pulmonary opacities remained radiologically stable under tafamidis therapy at two months and one year; however, pulmonary outcomes and the expected time course under TTR-targeted therapy remain uncertain. Accumulation of similar cases and prospective longitudinal observation are needed to define optimal management strategies for pulmonary manifestations of ATTR.
